# MELD score and antibiotics use are predictors of length of stay in patients hospitalized with hepatic encephalopathy

**DOI:** 10.1186/1471-230X-14-185

**Published:** 2014-10-17

**Authors:** Valérie Martel-Laferrière, Caitlin Homberger, Kian Bichoupan, Douglas T Dieterich

**Affiliations:** Department of Medicine, Division of Liver Diseases, Icahn School of Medicine at Mount Sinai, One Gustave L. Levy Place, New York, NY 10029 USA; Département de microbiologie et maladies infectieuses et Centre de Recherche du Centre Hospitalier de l’Université de Montréal, 1058 St-Denis, Montréal, H2X 3J4 Qc Canada

**Keywords:** Hepatic encephalopathy, Length of stay, MELD score

## Abstract

**Background:**

Hepatic encephalopathy (HE) represents a significant burden to the healthcare system. The aim of this study was to determine factors influencing the hospital length of stay among patients hospitalized with HE.

**Methods:**

A data warehouse query was performed to identify 316 patients with a first hospitalization during which HE occurred, between April 2010 and February 2012. Baseline and hospitalization characteristics were collected with IRB approval. A negative binomial multivariable model was used to control for potential confounders on the length of hospitalization.

**Results:**

Median age was 59 years, and 60.4% of admitted patients were male. The median MELD score was 22 (IQR: 17-28). Median length of stay was 8 days (IQR: 3.25-14.25). After controlling for MELD score, female gender (2.2 days; p = 0.04), being initially admitted for a reason other than HE (liver-related: 7.6 days; p < 0.01 and non liver-related 10.7 days; p < 0.01) and receiving antibiotics other than rifaximin (10.5 days; p < 0.01) were associated with longer length of stay whereas hepatitis C (-3.1 days; p < 0.01) was associated with a shorter length of stay.

**Conclusions:**

MELD score, gender, use of antibiotics other than rifaximin, reason for admission and hepatitis C are predictors readily available in clinic that can help identify patients at risk for longer length of stay.

## Background

Hepatic encephalopathy (HE) is a common complication of chronic liver disease, occurring in 30-45% of patients with cirrhosis [1, 2]. Hepatic encephalopathy is a spectrum of neuropsychiatric manifestations ranging from psychomotor difficulties to altered consciousness and even coma [3].

Each year HE is responsible for 0.33% of all hospitalizations, due to the susceptibility of a large population of persons with chronic liver disease, which comprises some 5.5 million people in the United States alone [2, 4]. HE is an expensive disease. Among hepatitis C (HCV) infected patients, HE costs are estimated at $16,430 for the first year and $3,810 in the subsequent years [5]. For those requiring hospitalization, length of stay is long, lasting 8.5 days on average, and costs approximately $63,000 per case [2]. HE-related hospitalizations are responsible for approximately 7.2 billion dollars in direct costs annually [2]. Fuelled by the aging of the HCV population and the growing incidence of non-alcoholic fatty liver disease, recent trends in the costs of HE-related hospitalization will likely continue.

To better understand the factors underlying the upward trend of hospitalizations and associated costs, this retrospective chart review investigates the length of stay of patients hospitalized with hepatic encephalopathy at a large academic tertiary care center.

## Methods

This is a retrospective chart-review study conducted at Mount Sinai Medical Center, a large tertiary care center. The Icahn School of Medicine at Mount Sinai Institutional Review Board approved the study (GCO #12-0998). A cohort of patients potentially hospitalized with hepatic encephalopathy was collected through a query of the electronic medical record using the ICD-9 codes 570.X, 572.2, 348.3, 348.31, 348.39 and 291.2. The charts of identified patients were reviewed to confirm study eligibility. Patients included were adults hospitalized between April 1^st^ 2010 and February 28^th^ 2012 for hepatic encephalopathy or for which hospitalization for another reason was complicated by hepatic encephalopathy. Children with hepatic encephalopathy and adults with metabolic encephalopathy unrelated to liver diseases were excluded. One event per patient was recorded. If more than one event occurred, the first event during the study period was recorded. Data were collected from electronic discharge summaries that included a full history, a list of interventions, lab results, medication profiles and discharge orders.

Patients could present with more than one baseline liver disease. HE hospitalizations were those for which HE was one of the primary diagnoses in the discharge summary. Liver-related hospitalizations were those for which a cirrhosis complication such as ascites or variceal bleeding was the primary diagnosis whereas non liver-related hospitalizations encompassed a large array of reasons of hospital admission such as infection or trauma. HE was not part of the primary diagnosis, but occurred during the hospitalization when the patient was categorized as “liver-related” or “non-liver related”. Length of stay was calculated from the day of admission to the day of discharge. If the patient was transferred from another hospital, the days spent in the other hospital were also counted. Thirty-day readmissions were calculated from the day of discharge. Patients who died during the first admission were excluded from the re-admission analysis. Model for End-Stage Liver Disease (MELD) score used was the crude MELD score based only on laboratory values (creatinine, International Normalized Ratio (INR) and bilirubin). Choice of treatment was up to the discretion of the clinician as Mount Sinai hospital does not have an HE protocol.

Descriptive statistics were used to investigate the outcomes of hospitalization. Factors influencing the length of hospitalization were identified with a univariable negative binomial regression model. A negative binomial regression was used due to over dispersion. All factors assessed were selected a priori. To control for potential confounders on the length of hospitalization, a multivariable negative binomial regression model was built. All variables with a p-value of <0.10 in univariable analysis were assessed in a multivariable model. Variables with a p-value less than 0.05 were retained in the final model. A second negative binomial model that excluded patients who died during the hospitalization, was created. A p-value less than 0.05 was considered statistically significant. Data were analyzed in SAS version 9.3.

## Results

### Baseline characteristics

The medical record query lead to the identification of 502 potential patients, of which 316 were confirmed to fit the inclusion criteria after chart review. The main reasons for exclusion were: age <18, or hospitalization for metabolic encephalopathy not related to liver disease.

Table 1 presents the baseline characteristics of the patients. The median age was 59 (IQR: 53-65), more patients were male than female (191, 60.4%) and a third were Caucasian (107, 33.9%). MELD scores were high with a median of 22 (IQR: 17-28). Hepatitis C was the most common underlying liver condition (110 patients, 35%) followed by alcohol (98, 31%). Only 15 patients (4.7%) had a transjugular intrahepatic portosystemic shunt (TIPS). For more than half the cohort, the hospitalization recorded was not the first episode of HE (161/289; 55.7%).

**Table 1:** **Baseline and hospitalization characteristics**

Patients characteristics	N = 316
Age, median (IQR)	59 (53-65)
Gender (male; %)	191 (60.4%)
Race (%)	
• Caucasian	107 (33.9%)
• African American	42 (13.3%)
• Asian	15 (4.7%)
• Hispanic	91 (28.8%)
• Unknown	61 (19.3%)
Baseline liver disease (%)	
• Hepatitis B	10 (3.2%)
• Hepatitis C	110 (35%)
• Alcohol	98 (31%)
• Non-alcoholic fatty liver disease	31 (10%)
• Autoimmune hepatitis	10 (3%)
• Primary biliary cirrhosis/Primary sclerosing cholangitis	10 (3%)
• Hemochromatosis	6 (2%)
• Post-liver complication	9 (3%)
• Cryptogenic/unspecified	58 (19%)
Presence of TIPS (%)	15 (4.7%)
Prior history of HE (%)	161/289 (55.7%)
MELD, median (IQR)	22 (17-28)
Primary reason of admission	
• HE	198 (62.7%)
• Liver related-complication	88 (27.8%)
• Non-liver related	30 (9.5%)
Acute hepatitis (%)	32 (10.1%)
Treatment	
• Lactulose alone	61 (19.3%)
• Lactulose + rifaximin	240 (75.9%)
• Rifaximin alone	10 (3.2%)
• Other^a^	5 (1.6%)
Use of antibiotics other than rifaximin during the admission (%)	181 (57.3%)
Length of stay, days, median (IQR)	8 (3.25-14.75)

^a^Metronidazole, neomycin, supportive

Hepatic encephalopathy was the main reason for hospital admission for 198 patients (62.7%). Of the remaining patients, 88 patients (27.8%) were hospitalized for a liver-related reason and 30 patients (9.5%) for a non liver-related reason. A small number of patients had acute hepatitis (32; 10.1%) or spontaneous bacterial peritonitis (34; 10.8%) during the hospitalization. Most patients received a combination of rifaximin and lactulose (240; 75.9%).

### Hospitalization outcomes

Sixty-six patients (20.9%) died during the initial hospitalization. Of the 250 patients discharged from their first hospitalization, 96 (38.4%) were readmitted within 30 days and an additional 34 patients were readmitted between 31 and 90 days for a total of 130 patients (52%) readmitted within 90 days. Overall, only 38% of the patients had a favourable outcome after their initial admission, meaning they did not die in the hospital or were not readmitted within 90-days.

### Predictors of length of hospitalization

The median length of stay was 8 days (IQR: 3.3-14.8 days), but the data contained outliers such as one hospitalization of 113 days. The median length of stay for various subgroups can be seen in Table 2. In univariable analysis, females, patients with acute hepatitis, patients with no prior history of HE or TIPS, and patients who required antibiotics other than rifaximin were more likely to have a longer length of hospitalization (Table 3). The baseline liver disease and the reason for admission also significantly influenced the duration of hospitalization.

**Table 2:** **Length of hospitalization**

Patients characteristics	Length of hospitalization in days (IQR)
Gender	
-Male	6.0 (3.0-13.0)
-Female	10.0 (4.0-17.0)
Age	
≤ 29	9.0 (5.0-47.0)
30-49	9.5 (4.0-16.0)
50-69	7.0 (3.0-15.0)
≥70	9.0 (3.0-13.0)
MELD score	
≤ 10	8.0 (3.0-21.0)
11-20	4.0 (2.0-10.0)
21-30	10.0 (5.0-16.0)
31-40	11.0 (6.0-27.0)
> 40	10.5 (5.5-24.5)
Hepatitis B	7.5 (2.0 – 11.0)
Hepatitis C	5.0 (3.0 -10.0)
Alcohol	9.0 (4.0 – 17.0)
Non-alcoholic fatty liver disease	7.0 (4.0 – 15.0)
Autoimmune hepatitis	11.5 (8.0 – 17.0)
PBC/PSC	7.5 (2.0 – 20.0)
Hemochromatosis	10.5 (10.0 – 11.0)
Post-liver transplant complication	12.0 (5.0 – 13.0)
Cryptogenic/unspecified	10.0 (5.0 – 24.0)
Reason of hospitalization	
-HE	5.0 (3.0-11.0)
-Liver-related	13.0 (7.0-22.8)
-Non liver-related	13.0 (7.8-24.0)
Acute hepatitis	14.0 (10.3-23.8)
TIPS	4.0 (1.0-14.0)
Prior HE	5.0 (3.0-12.0)
Rifaximin	8.0 (3.0-15.0)
Use of other antibiotics	11.0 (5.0-21.0)

**Table 3:** **Predictors of length of hospitalization**

**A. Overall cohort**				
	**Univariable analysis**		**Multivariable analysis**	
	**Change in length of stay (days)**	**p-value**	**Change in length of stay (days)**	**p-value**
Gender (female)	5.1	<0.01	2.2	0.04
Age			.	.
≤29	Reference	.		
30-49	-10.2	0.19		
50-69	-12.5	0.06		
≥70	-13.8	0.04		
MELD score				
≤10	Reference	.	Reference	.
11-20	-6.9	0.01	-5.4	<0.01
21-30	-1.8	0.59	-3.8	0.02
31-40	3.1	0.48	-2.9	0.15
>40	4.9	0.38	-2.5	0.33
Hepatitis B	-0.2	0.95	.	.
Hepatitis C	-6.1	<0.01	-3.1	<0.01
Alcohol	1.0	0.53	.	.
Non-alcoholic fatty liver disease	1.3	0.60	.	.
Autoimmune hepatitis	-1.4	0.73	.	.
PBC/PSC	3.1	0.50	.	.
Hemochromatosis	-2.1	0.67	.	.
Post-liver transplant complication	3.2	0.51	.	.
Cryptogenic/unspecified	6.1	<0.01	.	.
Reason of hospitalization				
-HE	Reference	.	Reference	.
-Liver-related	9.8	<0.01	7.6	<0.01
-Non liver-related	12.9	<0.01	10.7	<0.01
Acute hepatitis	9.7	<0.01	.	.
TIPS	-3.5	<0.01	.	.
Prior HE	-3.7	0.01	.	.
Rifaximin	1.9	0.28	.	.
Use of other antibiotics	10.2	<0.01	10.5	<0.01
**B. Survivors cohort**				
	**Univariable analysis**		**Multivariable analysis**	
	**Change in length of stay (days)**	**p-value**	**Change in length of stay (days)**	**p-value**
Gender (female)	5.5	<0.01	3.4	0.01
Age				
≤29	Reference	Ref	.	.
30-49	1.5	<0.01		
50-69	3.2	<0.01		
≥70	5.1			
MELD score				
≤10	Reference	Ref	Reference	Reference
11-20	-8.0	<0.01	-6.6	<0.01
21-30	-2.7	0.41	-5.1	<0.01
31-40	5.2	0.33	2.7	0.38
>40	15.6	0.13	3.0	0.52
Hepatitis B	-4.1	<0.01	.	.
Hepatitis C	-6.1	<0.01	-3.9	<0.01
Alcohol	3.2	0.04	.	.
Non-alcoholic fatty liver disease	2.7	0.30	.	.
Autoimmune hepatitis	-1.4	0.74	.	.
PBC/PSC	-8.2	0.01	-8.3	<0.01
Hemochromatosis	-0.8	0.86	.	.
Post-liver transplant complication	1.9	0.72	.	.
Cryptogenic/unspecified	6.0	0.01	.	.
Reason of hospitalization				
-HE	Reference	Ref	Reference	Ref
-Liver-related	21.8	<0.01	10.3	<0.01
-Non liver-related	27.7	<0.01	15.9	<0.01
Acute hepatitis	16.0	<0.01	.	.
TIPS	-6.8	<0.01	-4.5	0.04
Prior HE	-2.9	0.04	.	.
Rifaximin	-0.45	0.81	.	.
Use of other antibiotics	9.4	<0.01	9.9	<0.01

In multivariable analysis, we found the change in length of stay to be associated with female gender (2.2 days; p = 0.04), primary reason of hospitalization other than HE (liver-related: 7.6 days; p < 0.01 and non liver-related: 10.7 days; p < 0.01) and use of antibiotics other than rifaximin (10.5 days; p < 0.01) after controlling for MELD score. Additionally HCV-infected patients had shorter length of stay compared to patients without HCV (-3.1 days; p < 0.01) after controlling for MELD score.

When we excluded patients who died during the hospitalization, the median length of stay was 6 days (IQR: 3-13 days). As in the overall cohort, female gender, MELD score, primary reason of hospitalization other than HE and hepatitis C significantly influenced the length of stay. In addition, TIPS (-4.5 days; p < 0.04) and primary biliary cirrhosis/primary sclerosing cholangitis (-8.3 days; p < 0.01) were associated with a shortened length of stay.

## Discussion

Overall, clinical outcomes following a hospitalization for hepatic encephalopathy in a tertiary care liver transplant center were discouraging. Hospitalizations were long with a median of 8 days but a maximum up to 113 days and 33 patients (10.4%) hospitalized for longer than one month. In-patient mortality and 30-day readmissions were also frequent (20.9% and 38.6%, respectively). These numbers are slightly higher than previously reported mortality rates of 14.1-15.6% and 30-day readmission rates of 20% [2, 6].

We identified potential predictors of longer length of stay in our patient population. As expected, the length of stay in patients was negatively associated with MELD. Overall, patients with lower MELD had shorter length of stay, except for patients with MELD <10. HE is rare in patients with low MELD and hospitalization length may have been driven by another cause than HE.

To our knowledge, no other studies on the association between length of stay and infections or antibiotic use have been published to date. Although antibiotic use is likely a marker of sicker patients, it remains significant even after adjustment for MELD score. The impact of antibiotics on length of stay could originate from two pathways: a modification of the gut flora induced by the antibiotics or as a surrogate marker for infections. The latter is more probable as infection is common in patients with advanced liver disease and is a frequent trigger of HE [7].

In the past, infections have been associated with poor outcomes in patients with HE. Hung *et al*. retrospectively looked at 4,150 patients with HE and cirrhosis [8]. In a Cox proportional-hazards regression model, they found a significant association between the presence of a co-existing infection and 30-day, 30-90-day and 90-day-1 year mortality (HR: 1.66 (95% CI: 1.32-1.94), 1.51 (95% CI 1.23-1.85), 1.34 (95% CI 1.13-1.58), respectively) while adjusting for age, gender, HCV, variceal bleeding, ascites, alcoholism, acute and chronic renal failure and peptic ulcer bleeding [8]. In a recent prospective double-blind randomized controlled trial comparing lactulose with placebo to lactulose with rifaximin, higher baseline leukocyte count was associated with non-response to treatment [9]. Similarly, Sharma *et al*. found higher leukocyte counts among non-lactulose responders than among responders, but they did not find an association between infection and non-response except for the occurrence of spontaneous bacterial peritonitis and related leukocytes elevation to systemic inflammation of patients with advanced liver disease [10]. Shawcross *et al*. did not find any difference in survival between patients with and without positive cultures when they looked at 100 patients admitted to the intensive care unit for HE [11].

Admission for HE was associated with a shorter length of stay. It could be hypothesized that HE, as well as possible precipitants, were rapidly identified and treated in these patients. Similarly, among survivors, patients with TIPS had shorter length of stay. As HE is a frequent complication of TIPS (median cumulative 1-year incidence: 10-50%), it was likely sought after and managed more aggressively in these patients [12].

Patients with HCV liver disease had a shorter length of stay than patients with non-HCV liver disease. The reason for this observation is unclear. One explanation could be a slower and often more predictable progression of HCV-induced liver disease compared to other liver conditions. This is also supported by the fact that patients with primary biliary cirrhosis/primary sclerosing cholangitis, also slowly progressing diseases, had shorter lengths of stay in the survivors multivariable analysis. Wiegand *et al*. also evoked a difference in disease progression to explain clinical differences observed in type of decompensation between patients with alcoholic vs. non-alcoholic liver diseases [13].

## Conclusions

Female gender, hepatitis C infection, reason of admission, MELD score and use of antibiotics are predictors of length of stay. Because these characteristics are available to the clinicians rapidly after patient admission, they can be used to target interventions for patients at high risk and reduce both length of stay and readmission. Even if limited by their retrospective nature, our data are derived from a large cohort of patients and include patients with advanced liver disease (median MELD of 22).

## Abbreviations

HCV: Hepatitis C
HE: Hepatic encephalopathy
TIPS: Transjugular intrahepatic portosystemic shunt
MELD: Model for End-stage Liver Disease
INR: International Normalized Ratio
PBC: Primary biliary cirrhosis
PSC: Primary sclerosing cholangitis
IQR: Interquartile range.
